# Supplementary material for: In Silico TRials guide optimal stratification of ATrIal FIbrillation patients to Catheter Ablation and pharmacological medicaTION: the i-STRATIFICATION study
Source: Europace. 2024 Jun 13;26(6):euae150. doi: 10.1093/europace/euae150 (PMC11184207; doi:10.1093/europace/euae150)
Supplement: euae150_Supplementary_Data [file euae150_supplementary_data.docx]

**SUPPLEMENTAL MATERIAL**

**i**n-**S**ilico **TR**ials guide optimal stratification of **AT**r**I**al **FI**brillation patients to **C**atheter **A**blation and pharmacological medica**TION**

The i-STRATIFICATION study

Albert Dasí^*^, Claudia Nagel, Michael T.B. Pope, Rohan S. Wijesurendra, Timothy R. Betts, Rafael Sachetto, Axel Loewe, Alfonso Bueno-Orovio, Blanca Rodriguez^*^

* Corresponding author:

Blanca Rodriguez: blanca.rodriguez@cs.ox.ac.uk

Albert Dasí: albert.dasiimartinez@cs.ox.ac.uk

Department of Computer Science, University of Oxford, Wolfson Building, Parks Road OX1 3QD Oxford (UK)

**SUPPLEMENTAL METHODS**

All files used to conduct simulations (i.e., bi-atrial meshes and configuration files with the exact set of parameters used) are publicly available at https://zenodo.org/records/10562550.

**Anatomical Variability**

A human bi-atrial statistical shape model^1^ was used to generate ten atrial anatomies spanning anatomical variability in clinical data. As illustrated in **Figure S1**, these bi-atrial anatomies covered the volumes associated clinically with atrial fibrillation (AF) recurrence. The ten volumetric bi-atrial geometries are publicly available on (https://zenodo.org/records/5004620), and their corresponding ZENODO identifier is included in **Table S1**.

**
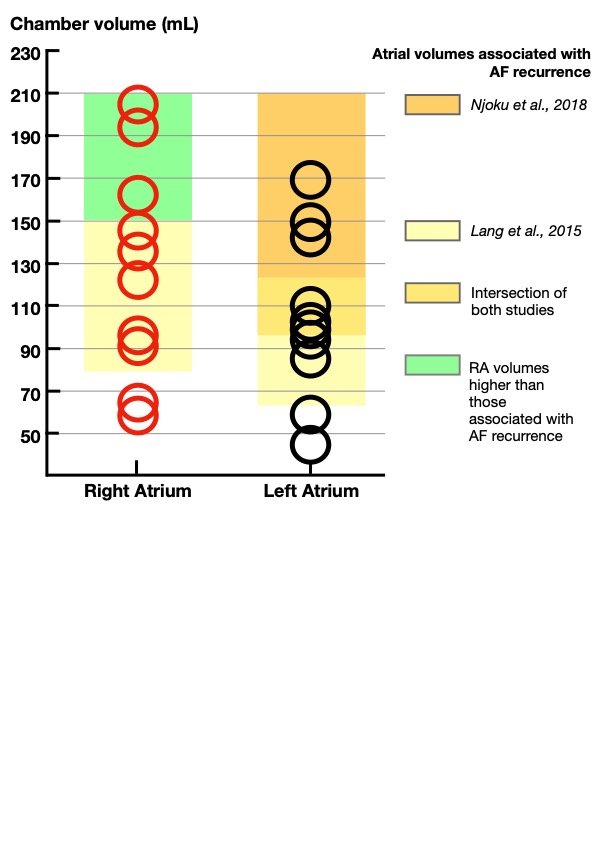
**

**Figure S1.** Right (RA) and left atrial volumes for the 10 atrial anatomies used in this study for multi-scale simulations. Comparison with the atrial volumes associated with atrial fibrillation (AF) recurrence clinically. The exact volumes for each anatomy are included in **Table S1**.

**Table S1.** Volume of the atrial anatomies considered in this study to conduct multi-scale simulations and corresponding ZENODO identifier. Comparison with the atrial volumes associated with AF recurrence clinically.

|  | | **ID on ZENODO** | **Atrial volume (mL)** | |
| --- | --- | --- | --- | --- |
|  |  |  | **Left atrium** | **Right atrium** |
| Atrial anatomy 1 (AA1) | | cn617_g078 | 102 | 58 |
| Atrial anatomy 2 (AA2) | | cn617_g149 | 59 | 64 |
| Atrial anatomy 3 (AA3) | | cn617_g016 | 44 | 91 |
| Atrial anatomy 4 (AA4) | | cn617_g055 | 146 | 96 |
| Atrial anatomy 5 (AA5) | | cn617_g112 | 85 | 122 |
| Atrial anatomy 6 (AA6) | | cn617_g104 | 110 | 135 |
| Atrial anatomy 7 (AA7) | | cn617_g095 | 169 | 145 |
| Atrial anatomy 8 (AA8) | | cn617_g065 | 94 | 162 |
| Atrial anatomy 9 (AA9) | | cn617_g014 | 142 | 194 |
| Atrial anatomy 10 (AA10) | | cn617_g082 | 99 | 205 |
| **Atrial volumes associated with**  **AF recurrence clinically** | Ref ^2^ |  | 65 – 112 | 80 – 148 |
|  | Ref ^3^ |  | 96 – 210 | – |

**Mesh transformation for finite volume method (FVM) simulations**

Each geometry in ZENODO^1^ consists of a VTK file that includes the bi-atrial anatomy with accurate fiber orientation, intra-atrial bridges and material tags in different atrial regions (i.e., right and left atria, right and left atrial appendage, pulmonary veins, superior and inferior cava vein, mitral and tricuspid valve, crista terminalis and pectinated muscles).

The VTK file (i.e., tetrahedral mesh) was converted to an ALG file (i.e., hexahedral mesh), suitable for the GPU solver MonoAlg3D, using the function **hexa-mesh-from-VTK**. This function and a detailed description of its usage can be found in the MonoAlg3D GitHub: (https://github.com/rsachetto/MonoAlg3D_C/wiki/Loading-a-VTU-mesh).

For this study, the hexahedral ALG meshes considered a spatial discretization of 400 μm.

**Electrophysiological variability: Population of human atrial cardiomyocyte models**

As described in^4^, an experimentally-calibrated in-silico population of human atrial cardiomyocyte models was developed using the CRN action potential model^5^ as baseline. The methodology has been extensively used in previous studies ^4^, and consists of scaling a subset of model parameters, which are thought to vary in the human population, over a specified range and retaining the models yielding simulated dynamics as in experiments. By using Latin hypercube sampling, every cardiomyocyte model of the population has a unique combination of parameters, which results in a unique electrophysiological profile and thus, action potential. It is commonly assumed that variability in cellular electrophysiology is mainly expressed at the level of the ion channel density^4^.

Building on previous populations studies and given the size of our simulation study, here, an initial population of 60 human atrial cardiomyocyte models was developed by scaling the maximal conductance (G) of key ionic currents over ±50%, including G_Kur_, G_Kr_, G_to_, G_K1_, G_CaL_, G_NaK_, and G_Na_. The generated population, called candidate population of cardiomyocyte models, undergoes a calibration step. The calibration is performed against human experimental data, and ensures that cardiomyocyte models accurately reproduce human electrophysiological properties. For this, action potential recordings from **persistent AF patients^6^** were compared to the in-silico signals. The experimentally-calibrated population comprises those cardiomyocyte models with action potential biomarkers within the ranges delimited by the minimum and maximum experimental value.

From the initial population of 60 cardiomyocytes models, 40 satisfied that all biomarkers (i.e., action potential duration at 90%, 50% and 20% of cellular repolarization, action potential amplitude, resting membrane potential and maximum upstroke velocity) were within the experimental ranges. These 40 cardiomyocyte models, which covered the wide variability in action potential biomarkers from AF patients, were kept for further analysis.

The scaling factors applied to the ionic current densities of the 40 atrial cardiomyocyte models are included in **Table S2**.

**Table S2.** Scaling factors used for developing the in-silico population of human atrial cardiomyocyte models. The population reproduced action potential biomarkers of persistent atrial fibrillation patients.

| **Atrial cardiomyocyte model (aCM model)** | **Scaling factors (no units) applied to the ionic current densities of the CRN model^5^** | | | | | | |
| --- | --- | --- | --- | --- | --- | --- | --- |
|  | G_Kur_ | G_Kr_ | G_to_ | G_K1_ | G_CaL_ | G_NaK_ | G_Na_ |
| **aCM model 1** | 0.7621 | 0.6003 | 0.9716 | 0.9698 | 0.5392 | 1.2136 | 1.3596 |
| **aCM model 2** | 0.7504 | 1.2355 | 0.7018 | 0.8079 | 0.7264 | 1.1587 | 0.6016 |
| **aCM model 3** | 0.9618 | 0.6763 | 1.4286 | 1.0725 | 0.5690 | 1.0276 | 0.6278 |
| **aCM model 4** | 0.6867 | 0.8860 | 1.4915 | 0.8829 | 0.5164 | 1.4273 | 0.5102 |
| **aCM model 5** | 0.6629 | 1.4646 | 0.5210 | 1.1076 | 0.8563 | 0.8431 | 1.1721 |
| **aCM model 6** | 0.7321 | 1.0148 | 0.9883 | 1.0247 | 1.0126 | 0.6828 | 1.3770 |
| **aCM model 7** | 0.9306 | 0.7603 | 0.8722 | 1.1521 | 0.9014 | 0.6048 | 0.7826 |
| **aCM model 8** | 1.0910 | 0.9791 | 0.5488 | 1.1223 | 0.6661 | 1.4412 | 0.8503 |
| **aCM model 9** | 1.2735 | 0.9331 | 1.4003 | 1.0963 | 1.1967 | 0.9270 | 1.0428 |
| **aCM model 10** | 1.0626 | 1.3265 | 1.1443 | 0.8156 | 0.5845 | 1.1001 | 1.0654 |
| **aCM model 11** | 1.0131 | 1.4589 | 1.0724 | 0.7910 | 1.3858 | 1.1989 | 0.9874 |
| **aCM model 12** | 0.9409 | 1.3687 | 0.7246 | 0.9926 | 1.0934 | 0.8998 | 1.4146 |
| **aCM model 13** | 1.2338 | 1.4070 | 1.4760 | 0.8378 | 1.0290 | 1.2434 | 1.4282 |
| **aCM model 14** | 0.5568 | 1.1475 | 1.0156 | 0.8991 | 1.3667 | 0.5746 | 0.7015 |
| **aCM model 15** | 1.1624 | 0.6497 | 0.8562 | 0.9085 | 0.6328 | 1.4164 | 0.5587 |
| **aCM model 16** | 1.0299 | 1.2460 | 1.3110 | 1.0664 | 0.8313 | 1.2923 | 1.2811 |
| **aCM model 17** | 0.8396 | 1.3125 | 0.6664 | 1.2132 | 0.7453 | 1.1233 | 0.5301 |
| **aCM model 18** | 1.3928 | 1.1939 | 1.4574 | 0.9511 | 0.9705 | 1.2603 | 1.0369 |
| **aCM model 19** | 1.2830 | 0.8181 | 1.0239 | 1.2453 | 1.1534 | 0.5458 | 0.9741 |
| **aCM model 20** | 1.3513 | 0.7267 | 0.5185 | 1.0047 | 0.6479 | 1.3131 | 0.8766 |
| **aCM model 21** | 0.6079 | 1.2794 | 0.7975 | 1.2292 | 1.2245 | 0.9092 | 0.8853 |
| **aCM model 22** | 1.2124 | 0.8523 | 1.2826 | 1.0386 | 0.9523 | 1.0651 | 0.9224 |
| **aCM model 23** | 0.8656 | 1.0386 | 0.7663 | 1.2397 | 1.2159 | 1.4614 | 1.0830 |
| **aCM model 24** | 0.8137 | 0.5142 | 1.1298 | 1.0473 | 1.0723 | 1.3574 | 0.9466 |
| **aCM model 25** | 0.5210 | 1.1757 | 1.1077 | 0.8224 | 1.3387 | 1.4956 | 0.6640 |
| **aCM model 26** | 1.1030 | 1.4856 | 0.6833 | 0.9106 | 0.8714 | 1.3734 | 0.8180 |
| **aCM model 27** | 1.3045 | 0.8259 | 1.3998 | 1.1198 | 0.7662 | 0.9532 | 1.0095 |
| **aCM model 28** | 0.5940 | 0.9057 | 0.6496 | 0.9894 | 0.7099 | 0.9827 | 0.9188 |
| **aCM model 29** | 0.7025 | 1.2128 | 1.2323 | 1.1833 | 1.4857 | 1.0465 | 0.5837 |
| **aCM model 30** | 0.6440 | 1.3528 | 1.1657 | 0.9376 | 1.4259 | 0.9697 | 1.2034 |
| **aCM model 31** | 1.4437 | 0.7812 | 0.7444 | 1.0811 | 1.1017 | 0.6698 | 0.7655 |
| **aCM model 32** | 0.8400 | 1.0911 | 1.3257 | 1.1454 | 0.8059 | 1.0921 | 0.6533 |
| **aCM model 33** | 1.3791 | 0.8778 | 1.2576 | 1.1339 | 0.9973 | 0.8616 | 1.2558 |
| **aCM model 34** | 0.7953 | 1.0427 | 0.5935 | 0.9469 | 1.3067 | 0.5802 | 0.7532 |
| **aCM model 35** | 1.1314 | 1.1193 | 1.0427 | 1.1787 | 1.4125 | 0.8289 | 0.7308 |
| **aCM model 36** | 0.6323 | 0.9572 | 0.8152 | 1.1604 | 1.4520 | 1.2354 | 1.1120 |
| **aCM model 37** | 1.3375 | 0.7139 | 0.9394 | 0.8701 | 0.6808 | 0.7640 | 0.6850 |
| **aCM model 38** | 0.8817 | 1.3949 | 0.9547 | 0.9778 | 0.6177 | 1.1752 | 1.3395 |
| **aCM model 39** | 1.1482 | 1.2872 | 1.0914 | 1.0529 | 0.8907 | 1.3276 | 1.2363 |
| **aCM model 40** | 1.4638 | 1.4205 | 1.3410 | 0.9297 | 1.2568 | 1.3909 | 1.1526 |

**Electrophysiological heterogeneities in different atrial regions**

Each of the 40 cardiomyocyte models, representative of the right atrial tissue, was scaled to reflect electrophysiological heterogeneities in six atrial regions (i.e., left atrium, crista terminalis, pectinate muscles, left atrial appendage and atrio-ventricular rings). The scaling factors are available in **Table S3**. The resulting seven action potential models (i.e., the original cardiomyocyte model and the scaled versions) constituted an ionic current profile. Every virtual patient presented a unique combination of ionic current profile and atrial anatomy.

Regional heterogeneities in conduction velocity and anisotropy were also included, as described in **Table S3**. The longitudinal and transversal conductivity values used in MonoAlg3D to achieve the conduction velocities illustrated in **Table S3** are provided in **Table S4**. Other parameters of the monodomain equation, such as the surface-volume ratio and the membrane capacitance, were defined as β =1400 cm^-1^ and C_m_ = 1 μF∕cm^2^, respectively. The values in **Table S4** were fixed for all simulations and thus, inter-patient differences in conduction velocity were only the result of different ionic current profiles (see below).

**Table S3.** Regional electrophysiological heterogeneities in ionic current densities and conduction velocity, adapted from^7^. The original cardiomyocyte model was included in the right atrial tissue.

| **Region** | **Conductivity ratio**  **Transversal-Longitudinal** | **CV (cm/s)** | **I_to_** | **I_CaL_** | **I_Kr_** |
| --- | --- | --- | --- | --- | --- |
| Right atrium | 1:2 | 80 | 1 | 1 | 1 |
| Left atrium | 1:2 | 80 | 1 | 1 | 1.6 |
| Sinoatrial node | 1:1 | 42 | 1 | 1 | 1 |
| Crista terminalis | 1:10 | 157 | 1.35 | 1.6 | 0.9 |
| Pectinate muscles | 1:2 | 133 | 1.05 | 0.95 | 0.9 |
| Bachmann’s bundle | 1:2 | 133 | 1 | 1 | 1 |
| Left atrium appendage | 1:2 | 80 | 0.65 | 1.05 | 2.75 |
| Atrio-ventricular rings | 1:2 | 80 | 1.05 | 0.65 | 3 |

**Table S4.** Conductivity values used in MonoAlg3D.

| **Region** | **Longitudinal conductivity** (mS/μm) | **Transversal conductivity** (mS/μm) |
| --- | --- | --- |
| Right atrium | 0.000641 | 0.00032 |
| Left atrium | 0.000641 | 0.00032 |
| Sinoatrial node | 0.000210 | 0.00020 |
| Crista terminalis | 0.002000 | 0.00020 |
| Pectinate muscles | 0.001600 | 0.00080 |
| Bachmann’s bundle | 0.001600 | 0.00080 |
| Left atrium appendage | 0.000641 | 0.00032 |
| Atrio-ventricular rings | 0.000641 | 0.00032 |

**Calibration of the tissue conductivity to obtain accurate conduction velocities**

To compute conduction velocities, we employed a cable tissue of 5.00×0.04×0.04 cm^3^, with a spatial discretization of 400 µm (same as in multi-scale simulations). Five consecutive beats were paced at one side of the cable at different cycle lengths. Cardiomyocyte models were previously paced for 50 beats in a single-cell environment, so they could adapt to the different cycle lengths. Two nodes in the mesh, located at 2 and 3 cm from the stimulation point, were chosen to compute the conduction velocity, in order to avoid boundary effects. The conduction velocity was therefore calculated as the distance between these points (i.e., 1 cm) over the time difference at which the cells were depolarized. A threshold of –50 mV was selected to denote cell depolarization.

In the baseline CRN action potential model^5^ (i.e., with no ionic current density variation) the longitudinal conductivity was set to 0.000641 mS/μm to obtain a baseline plane wave velocity of 80 cm/s in the bulk tissue (i.e., right and left atrium), as shown in **Table S3–S4**.

After considering variability in the ionic current profile, especially in I_Na_ density (i.e., main ionic current influencing conduction velocity), the population of virtual patients had a longitudinal conduction velocity in the healthy bulk tissue (i.e., tissue not defined as low voltage areas, LVA) ranging between 72.3 and 110.9 cm/s and between 58.8 and 102.5 cm/s when paced at 1 Hz and 4 Hz, respectively. Moreover, a similar range of variability was observed in the remaining atrial regions which baseline conduction velocity was different from 80 cm/s (e.g., crista terminalis). This is in accordance with values of conduction velocity reported clinically^8^.

**Structural variability**

**Low voltage areas (LVA) registration:** The electro-anatomical maps of 20 (76% persistent, 41% female) AF patients were obtained at the John Radcliffe Hospital in Oxford, by Dr Rohan S. Wijesurendra, Dr Michael T.B. Pope and Dr Timothy R. Betts. All studies were conducted according to the principles of the Declaration of Helsinki, and all patients gave written informed consent.

High density mapping of the atria was performed using an Abbott Advisor HD grid (SE) catheter while pacing at the coronary sinus at a cycle length of 800 ms. The patient electro-anatomical maps were registered to the endocardial surface of the bi-atrial statistical shape model, through a rigid and non-rigid registration.

The registration was performed using MeshMonk^9^, which is an open-source toolbox implemented in MATLAB. The program orients, repositions, and scales a template surface (i.e., in this case the patients’ endocardial surface) to a target surface (i.e., the endocardial surface of the mean shape model), during a rigid registration step. Subsequently, the template surface is further transformed to fit the specific shape of the target surface, using a non-rigid deformation. To perform the rigid and non-rigid registration, common landmarks need to be placed in the target and template surfaces. For the registration of the patient-specific maps, nine landmarks were placed in the left atrium, namely, the intersection of the four veins with the left atrial posterior wall, the tip of the appendage and four equally-spaced point around the mitral ring. Similarly, 7 points were placed in the right atrial surfaces: the intersection of the superior and inferior cava vein with the venous portion of the chamber, the tip of the appendage and four equally-spaced point around the tricuspid ring.

After registration, the patients’ voltage data were subsequently interpolated to the target surface using the nearest neighbor algorithm.

**Modelling and simulation of LVA:** LVAs were simulated as regions of 30% decreased longitudinal conductivity, increased anisotropy (i.e., 8:1 longitudinal to transversal conductivity ratio) and 50%, 40% and 50% reductions in I_CaL_, I_Na_ and I_K1_, respectively^10^. This remodeling was applied on top of the individual electrophysiological properties of each virtual patient. Thus, the 30% reduced longitudinal conductivity and the 40% I_Na_ reduction had a different effect in those virtual patients with the slowest and fastest conduction velocity (i.e., velocity in the bulk tissue of 58.8 and 102.5 cm/s, respectively). After applying these changes in LVA, the slowest longitudinal and transversal conduction velocities observed in LVA were 50.1 cm/s and 10.5 cm/s, respectively.

**Convergence analysis**

The slowest conduction velocity was obtained for the atrial cardiomyocyte #2 (**Table S2**). This ionic current profile presented a low I_Na_ multiplier (i.e., 0.6016) which, in control conditions, yielded longitudinal and transversal conduction velocities of 72.3 and 49.0 cm/s, respectively (**Figure S2**). After applying the LVA remodeling, these values decreased to 50.1 cm/s and 10.5 cm/s (**Figure S2**).

**Figure S2 - 1D analysis** illustrates the impact of mesh resolution on the conduction velocity of this atrial cardiomyocyte model, both in control and under LVA remodeling. In control conditions, a spatial resolution of 400 μm provided longitudinal and transversal conduction velocity values with less than 10% error compared to the finest spatial resolution tested (i.e., 25 μm element length). Accordingly, we expect all our simulations in structurally healthy atria to be absent of any significant numerical artifacts.

The above observations also held for the longitudinal conduction velocity under LVA remodeling. However, the slower transversal conduction velocity required in this case a finer mesh resolution of 100 μm to attain numerical errors below 10% (**Figure S2 - 1D analysis**). To assess whether a coarser mesh resolution of 400 μm as the one used in our study could have produced spurious wave break-ups resulting from numerical artifacts, re-entry dynamics were analyzed using the atrial cardiomyocyte #2 under LVA remodeling. **Figure S2 - 2D analysis** illustrates snapshots of a re-entry induced in a 2D tissue (10×10 cm^2^) with three different spatial resolutions: 100, 200, and 400 μm. While wavefront speeds were slower at the coarsest mesh resolution, all three resolutions exhibited qualitatively similar re-entry dynamics. In particular, neither numerical conduction block nor artifactual wave-breakups were observed in any scenario.

For completeness, **Figure S3** expands our analysis by using atrial cardiomyocyte #13, characterized by the highest I_Na_ density (**Table S2**) and yielding a faster conduction velocity in all conditions (**Figure S3**). In this case, smaller errors were obtained for a mesh resolution of 400 μm, especially for the transversal conduction velocity under LVA remodeling. Moreover, re-entry dynamics were almost identical when using 100 or 400 μm. Altogether, while acknowledging that quantitative differences in re-entry dynamics may arise in LVA at feasible resolutions for our large-scale simulation study, we expect our results in structurally remodeled atria to be minimally affected by numerical artifacts.

**Figure S2. Convergence analysis in 1D tissue.** Impact of mesh resolution on the longitudinal and transversal conduction velocity, in control conditions and under low voltage areas (LVA) remodeling, using the atrial cardiomyocyte model #2 (**Table S2**), characterized by low I_Na_ density. The horizontal lines represent 10% variation from the conduction velocity obtained with the finest mesh resolution (i.e., 25 μm edge length). **Re-entry dynamics in anisotropic 2D tissue.** Consecutive snapshots of the transmembrane voltage obtained in a 2D tissue of 10×10 cm^2^ with three different mesh resolutions: 100, 200, and 400 μm. A S1-S2 stimulation protocol is employed, applying the S2 stimulus 300 ms after the last S1.

**Figure S3. Convergence analysis in 1D tissue.** Impact of mesh resolution on the longitudinal and transversal conduction velocity, in control conditions and under low voltage areas (LVA) remodeling, using the atrial cardiomyocyte model #13 (**Table S2**), characterized by high I_Na_ density. The horizontal lines represent 10% variation from the conduction velocity obtained with the finest mesh resolution (i.e., 25 μm edge length). **Re-entry dynamics in anisotropic 2D tissue.** Consecutive snapshots of the transmembrane voltage obtained in a 2D tissue of 10×10 cm^2^ with three different mesh resolutions: 100, 200, and 400 μm. A S1-S2 stimulation protocol is employed, applying the S2 stimulus 300 ms after the last S1.

**AF induction**

The AF induction protocol considered arrhythmia as already present in the tissue, and assessed AF maintenance under different patient characteristics. For this, spiral wave re-entries were imposed as the initial conditions of the simulation^12^,^13^. Three spiral waves were applied in each atrial chamber. In the left atrium, one spiral wave was induced in the anterior wall, one in the posterior, and another in the inferior wall. In the right atrium, two spiral waves were induced in the venous portion, one around the proximity of the superior cava vein and one close to the inferior cava vein, and a third one in the anterior wall. The direction of rotation was clockwise for two spiral waves in each chamber and counter-clockwise in the third one, ensuring that adjacent re-entries rotated with opposing phase^12^.

The complete initial state vectors for imposing AF in the 10 atrial meshes, in control conditions and after the application of the nine different ablation strategies, are publicly available at https://zenodo.org/records/10562550.

**REFERENCES**

1. Nagel C, Schuler S, Dössel O, Loewe A. A bi-atrial statistical shape model for large-scale in silico studies of human atria: model development and application to ECG simulations. Medical Image Analysis. 2021;74:102210.
2. Lang RM, Badano LP, Mor-Avi V, Afilalo J, Armstrong A, Ernande L, et al. Recommendations for Cardiac Chamber Quantification by Echocardiography in Adults: An Update from the American Society of Echocardiography and the European Association of Cardiovascular Imaging. European Heart Journal - Cardiovascular Imaging. 2015 02;16(3):233-71.
3. Njoku A, Kannabhiran M, Arora R, Reddy P, Gopinathannair R, Lakkireddy D, Dominic P. Left atrial volume predicts atrial fibrillation recurrence after radiofrequency ablation: a meta-analysis. EP Europace. 2018; 20(1): 33–42.
4. Muszkiewicz A, Britton OJ, Gemmell P, Passini E, Sánchez C, Zhou X, et al. Variability in cardiac electrophysiology: Using experimentally-calibrated populations of models to move beyond the single virtual physiological human paradigm. Progress in Biophysics and Molecular Biology. 2016; 120(1–3): 115–127.
5. Courtemanche M, Ramirez RJ, Nattel S. Ionic mechanisms underlying human atrial action potential properties: insights from a mathematical model. American Journal of Physiology-Heart and Circulatory Physiology. 1998;275(1):H301-21.
6. Sánchez C, Bueno-Orovio A, Wettwer E, Loose S, Simon J, Ravens U, et al. Inter-Subject Variability in Human Atrial Action Potential in Sinus Rhythm versus Chronic Atrial Fibrillation. PLOS ONE. 2014 08;9(8):1-14.
7. Sánchez C, Bueno-Orovio A, Pueyo E, Rodriguez B. Atrial Fibrillation Dynamics and Ionic Block Effects in Six Heterogeneous Human 3D Virtual Atria with Distinct Repolarization Dynamics. Frontiers in Bioengineering and Biotechnology. 2017; 5: 1-13.
8. Mikhailov AV, Kalyanasundaram A, Li N, Scott SS, Artiga EJ, Subr MM, et al. Comprehensive evaluation of electrophysiological and 3D structural features of human atrial myocardium with insights on atrial fibrillation maintenance mechanisms. Journal of Molecular and Cellular Cardiology. 2021; 151: 56-71.
9. White JD, Ortega-Castrillón A, Matthews H, Zaidi AA, Ekrami O, Snyders J, et al. MeshMonk: open-source large-scale intensive 3D phenotyping. Scientific Reports. 2019; 9: 6085.
10. Azzolin L, Eichenlaub M, Nagel C, Nairn D, Sanchez J, Unger L, et al. Personalized ablation vs. conventional ablation strategies to terminate atrial fibrillation and prevent recurrence. EP Europace. 2023; 25(1): 211–222.
11. Nairn D, Eichenlaub M, Müller-Edenborn B, Huang T, Lehrmann H, Nagel C, et al. Differences in atrial substrate localization using late gadolinium enhancement-magnetic resonance imaging, electrogram voltage, and conduction velocity: a cohort study using a consistent anatomical reference frame in patients with persistent atrial fibrillation. EP Europace. 2023; 25(9): euad278.
12. Matene E, Jacquemet V. Fully automated initiation of simulated episodes of atrial arrhythmias. EP Europace. 2012; 14: v17–v24.
